# Morphine Stimulates Cell Migration of Oral Epithelial Cells by Delta-Opioid Receptor Activation

**DOI:** 10.1371/journal.pone.0042616

**Published:** 2012-08-10

**Authors:** Nada Charbaji, Monika Schäfer-Korting, Sarah Küchler

**Affiliations:** Institute of Pharmacy, Freie Universität Berlin, Berlin, Germany; Chang Gung University, Taiwan

## Abstract

Oral mucositis is one of the most common side effects of chemoradiation regimens and manifestation can be dose-limiting for the therapy, can impair the patient's nutritional condition and quality of life due to severe pain. The therapeutic options are limited; often only an alleviation of the symptoms such as pain reduction by using systemic opioids is possible. Stimulating opioid receptors on peripheral neurons and dermal tissue, potent analgesic effects are induced e.g. in skin grafted patients. Advantageous effects on the cell migration and, thus, on the wound healing process are described, too. In this study, we investigated whether opioid receptors are also expressed on oral epithelial cells and if morphine can modulate their cell migration behavior. The expression of the opioid receptors MOR, DOR and KOR on primary human oral epithelial cells was verified. Furthermore, a significantly accelerated cell migration was observed following incubation with morphine. The effect even slightly exceeded the cell migration stimulating effect of TGF-ß: After 14 h of morphine treatment about 86% of the wound area was closed, whereas TGF-ß application resulted in a closed wound area of 80%. With respect to morphine stimulated cell migration we demonstrate that DOR plays a key role and we show the involvement of the MAPK members Erk 1/2 and p38 using Western blot analysis.

Further studies in more complex systems *in vitro* and *in vivo* are required. Nevertheless, these findings might open up a new therapeutic option for the treatment of oral mucositis.

## Introduction

Oral mucositis (OM) is an acute inflammation and ulceration of the oral mucosa and often occurs as an adverse effect of chemo- and/or radiotherapy. The prevalence of OM strongly depends on the malign underlying disease and the required therapy regimen. About 30% of patients during or after chemotherapeutic treatment of many solid tumors and almost 100% of the patients undergoing a hematopoietic stem cell transplantation or radiotherapy of tumors in the head and neck area are affected [1], [2].

The occurrence of OM includes various symptoms beginning with slight redness up to deep ulcerations of the mucosa which is a dose-limiting factor for the chemotherapy, can impair the nutritional condition and liquid intake, affects the quality of life due to severe pain, and may result in serious clinical complications such as secondary fungal or viral infections. The patients experience OM as one of the most serious side effects of tumor therapy due to the severe pain which often results in a dropout or suboptimal dosing [3]. As a consequence the mortality of tumor patients with OM is increased. The clinical course of OM comprises five stages: Initiation, inflammation, aggravation, ulceration and finally healing [3]. Currently, complete prevention is not possible and the disease management is still complicated for both the patient and health provider as the therapeutic options are limited. General approaches include effective oral care (antiseptics etc.), topical mucosal protectants and dietary modifications. For the treatment of OM following hematopoietic stem cell transplantation palifermin, a recombinant keratinocyte growth factor, is approved. However, this only reflects 4% of the cases. Thus, the cornerstones of the therapy remain the use of topical anesthetics and for more severe cases the systemic use of analgesics, especially opioids [4]. Systemic application of opioids requires balancing the pain relief and the undesirable side effects such as nausea, vomiting, mental clouding, constipation and sedation [5], [6].

Therefore, local opioid application would be advantageous to reduce opioid-associated adverse effects. The rational basis for this approach is the expression of opioid receptors outside of the central nervous system on peripheral sensory neurons, tissues and cells such as keratinocytes and fibroblasts [6], [7], [8], [9], [10] and the induction of potent analgesic effects by activating these peripheral receptors [11], [12], [13]. Moreover, following topical application [14], [15], [16], [17], adverse effects are reduced. Additionally, opioids also modulate cell proliferation and survival (Chen, Law et al. 2008) and facilitate the wound healing and reepithelialization of skin wounds [9], [18] by stimulating keratinocyte migration [19], [20] as demonstrated repeatedly *in vitro* and *in vivo*. Furthermore, a functional role of opioids in the context of inflammation is well documented [11], [13].

Based on those results we investigated whether topically applied opioids - particularly morphine - might be a new therapeutic option for the treatment of OM with respect to pain relief and improved wound healing. Potent pain relief in patients suffering from OM was already shown when applying morphine locally as mouthwash [21]. In this study, we investigated whether opioid receptors are expressed on oral epithelial primary cells and cell lines and studied the effects of morphine on cell migration, viability and proliferation.

## Materials and Methods

### Chemicals

Morphine hydrochloride was purchased from Fagron (Barsbüttel, Germany), naloxone hydrochloride dihydrate, nor-Binaltorphimine dihydrochloride, naltrindole hydrochloride, DAMGO ([D-Ala2, N-Me-Phe4, Gly5-ol]-enkephalin acetate salt, DPDPE ([D-Pen2,D-Pen5]-enkephalin hydrate), 3-(4,5-dimethylthiazol-2-yl)-2,5-diphenyltetrazolium bromide (MTT), bovine serum albumin (BSA), U-69593 ((+)-(5α,7α,8β)-N-methyl-N-[7-(1-pyrrolidinyl)-1-oxaspiro[4.5]dec-8-yl]-benzeneacetamide, U0126 (1,4-Diamino-2,3-dicyano-1,4-bis(o-aminophenylmercapto)butadiene monoethanolate) and transforming growth factor-b1 (TGF-ß1) were obtained from Sigma-Aldrich (Munich, Germany), CTOP (H-D-Phe-Cys-Tyr-D-Trp-Orn-Thr-Pen-Thr-NH_2_) was purchased from Bachem (Bubendorf, Switzerland). Test substances were dissolved in phosphate-buffered saline (PBS, pH 7.4) with 0.4% BSA. U-69593 and U0126 were dissolved in dimethylsulfoxide (DMSO). TR146 cell line was obtained from the Imperial Cancer Research Technology (London, UK). Primary human oral keratinocytes (HOK) were purchased from Sciencell Research Laboratories (Carlsbad, CA, USA), the neuroblastoma cell line SHSY5Y was a gift from Prof. Dr. med. Christoph Stein (Charité Campus Benjamin Franklin, Berlin, Germany).

All solvents and diluents were purchased from Carl Roth (Karlsruhe, Germany).

### Cell Culture

TR146 cells, a human buccal tumour cell line, were maintained in 75 cm^2^ flasks (TPP Techno Plastic Products, Trasadingen, Switzerland) with Dulbecco's Modified Eagle's Medium/Nutrient Mixture F-12 (Sigma-Aldrich) supplemented with 10% fetal calf serum (FCS, Biochrom, Berlin, Germany), L-glutamine (5 mM) and penicillin/streptomycin (PAA Laboratories, Pasching, Austria). Cells were grown at 37°C and 5% CO_2_ and medium was changed every two or three days. At confluence, cells were split 1∶10 or 1∶15. Primary human oral keratinocytes (HOK) were cultured in 75 cm^2^ flasks pre-coated with 2 µg/cm^2^ of poly-L-lysine using oral keratinocyte medium. Medium was changed every two to three days until a confluence of 70%–80%. SHSY5Y cells was also cultured in 75 cm^2^ culture flasks and was grown in DMEM/HAM's F12 supplemented with 10% FCS, 1% penicillin/streptomycin, 1% glutamine und 10 mM non-essential amino acids solution (Biochrom).

Primary human keratinocytes (NHK) were isolated from juvenile foreskin after circumcision surgeries. Keratinocytes were grown in keratinocyte growth medium containing epidermal growth factor, insulin, gentamicin sulfate, amphotericin B, hydrocortisone and bovine pituitary extract (Lonza, Walkersville, MD, USA). Keratinocytes of the second or third passage were used for the experiments [22]. All cell lines were tested regularly for mycoplasma using Venor®Gem (Minerva Biolabs, Berlin, Germany) mycoplasma detection kit according to manufacture protocol.

### Reverse Transcription-PCR

RNA was isolated from TR146, HOK, SHSY5Y and NHK using NucleoSpin® RNA II kit (Macherey-Nagel, Düren, Germany) as described by the manufacturer. Total RNA amount and purity were determined using UV spectroscopy (wavelength setting: 260 nm and 280 nm) and gel electrophoresis. Prior to cDNA synthesis, the RNA samples were treated with DNase amplification grade I (Sigma-Aldrich, Steinheim, Germany), subsequently cDNA was generated using the FermentasAid™ First strand cDNA synthesis kit (Fermentas, St Leon-Rot, Germany). For relative quantification of opioid receptor expression RT-PCR was performed using a LightCycler 480 and the SYBR Green I Masterplus kit (Roche, Penzberg, Germany) according to manufacturer's instruction. Primer sequences are shown in table 1. Primers (TIB Molbiol, Berlin, Germany) were dissolved in molecular grade water to a final concentration of 10 µM. The mRNA expression levels of each of the targeted genes are presented as a ratio to the housekeeping gene YWHAZ. PCR product sizes were checked using a 2% agarose gel.

**Table 1:** Primer sequences and expected product size (bp) for the target and reference genes. Primer efficiency was >1.89, respectively.

Opioid Receptor (OR)	Primer sequences (5′→3′)	bp
DOR forward	ACCAGCACGCTGCCTTTCC	155
DOR reverse	ACAGCGATGTAGCGGTCAACAC	
MOR forward	TCCAGACTGTTTCTTGGCACTTC	130
MOR reverse	GAAGAGGTTGGGATACAGAACTCTC	
KOR forward	CGTCTGCTACACCCTGATGATC	64
KOR reverse	CTCTCGGGAGCCAGAAAGG	
YWHAZ forward	AGACGGAAGGTGCTGAGAAA	127
YWHAZ reverse	GAAGCATTGGGGATCAAGAA	

### Immunocytochemistry

To investigate opioid receptor expression on the protein level, immunocytochemistry was performed. TR146, HOK, SHSY5Y cells were fixed in 4% paraformaldehyde solution (in PBS, pH 7.4) for 20 min. Slides were washed in ice cold PBS and blocked with 1% BSA (Aurion, Wageningen, Netherlands) for 1 h at room temperature. Each slide was incubated with one of the primary antibodies anti-MOR (mu Opioid Receptor), anti-KOR (kappa Opioid Receptor) and anti-DOR (delta Opioid Receptor) (rabbit, Abcam, Cambridge, UK) at 4°C overnight, subsequently washed three times with PBST, followed by a one hour incubation with the fluorescein isothiocyanate (FITC) conjugated secondary anti-rabbit antibody (Abcam, Cambridge, UK). Afterwards, the slides were washed, covered with mounting medium DAPI (Dianova, Hamburg, Germany), and visualized using a Keyence digital microscope BZ-8000 (Keyence, Neu-Isenburg, Germany).

### *In Vitro* Wound Healing Assay

To investigate the effect of opioids on cell migration and wound closure of oral epithelial cells, the scratch assay was performed. Cells were seeded in six-well plates (TPP, Trasadingen, Switzerland) in a density of 2×10^5^ cells/well. After 48 h, a scratch was made through each well using a sterile pipette tip. Morphine (in PBS plus 0.4% BSA) was added in a concentration range of 1 nM to 10 µM. TGF-ß (1 ng/ml) served as positive control (for review, see [23]). Scratches were investigated under the microscope (magnification 100×) immediately after wounding and after cultivation in an incubator (37°C, 5% CO_2_) for 14 hours. Pictures were taken exactly at the same position before and after the incubation. To check for opioid-receptor mediated effects, a pre-incubation of the cells with the opioid receptor antagonist naloxone (10 µM for 1 h) was performed. In order to identify the opioid receptor being responsible for the cell migration enhancement, cells were also stimulated with DAMGO, DPDPE and U-69593 - MOR, DOR and KOR specific agonists - respectively. Additionally, prior to morphine stimulation we also pre-incubated the cells with selective MOR (CTOP), KOR (nor-Binaltorphimine dihydrochloride) and DOR (naltrindole hydrochloride) antagonists. For data evaluation, wound closure rate was calculated using the T scratch analysis software [24] which is based on image analysis technique and enables an automated calculation of the repair process.

### Knock Down of DOR, MOR and KOR using siRNA

To further elucidate the role of the opioid receptors on epithelial cell migration, TR146 cells were transfected with selective siRNAs for MOR (siRNA ID # s9871), DOR (siRNA ID # s9862), and KOR (siRNA ID # s9867) obtained from Ambion (Life Technologies, Darmstadt, Germany). For control, scrambled siRNA (Invitrogen, Carlsbad, CA, USA) was used. Prior to transfection, the siRNA was complexed with HiPerFect transfection Reagent (Qiagen, Hilden, Germany). 2×10^5^ cells/well were seeded in 6-well plates. The siRNA complex was added to a final concentration of 10 nM. Cells were incubated at 37°C, 5% CO_2_ for 48 h. Afterwards, RNA was isolated and RT-PCR was performed to assess the knock down efficiency.

After confirming sufficient knock down, the *in vitro* wound healing assay was performed (as described above).

### Cell Proliferation

The cell proliferation rate was assessed using Calbiochem®BrdU cell proliferation kit (Merck, Darmstadt, Germany). The proliferation kit detects 5-bromo-2′-deoxyuridine (BrdU) incorporation into cellular DNA during cell proliferation. Cells were seeded in 96-well plates. After cell attachment, they were stimulated with morphine in a concentration range from 1 nM to 100 µM. After 4 hours of stimulation 20 µl BrdU was added and the colored reaction product was quantified using a spectrophotometer (FLUOstar Optima, BMG LABTECH, Ortenberg, Germany), results were normalized to the untreated control.

### Cell Viability

For cell viability testing, the activity of the cellular mitochondrial dehydrogenase was determined by measuring MTT reduction and conversion into a blue formazan salt as described previously [25]. 1×10^4^ TR146 cells/well were seeded into 96-well. After 24 hours, the cells were stimulated with different concentrations of morphine for 14 h and 24 h at 37°C, respectively. Subsequently, 10 µl/well of MTT solution (5 mg/ml) were added. After 4 hours, the supernatants were removed, 50 µl of dimethylsulfoxide (DMSO) was added to dissolve the formazan salt and its optical density (OD) was measured using the FLUOstar Optima setting the absorbance to 540 nm [22]. Sodium dodecyl sulfate (0.01%) served as positive control. Each concentration was tested in triplicate and the experiments were repeated three times. A cell viability <70% predicts cytotoxic effects. The experiment was performed with the primary HOK, too.

### Determination of the Involvement of MAPK in Morphine Stimulated Cell Migration

25×10^4^ TR146 cells were seeded in six-well plates till confluence. To determine the involvement of Erk (Extracellular signal-regulated kinase) phosphorylation on morphine stimulated cell migration, cells were pre-incubated with 10 µM U0126 for 30 min. U0126 is a selective inhibitor of the protein kinases MEK1 and MEK2 which leads to the inhibition of the phosphorylation of Erk 1 and 2 [26]. Afterwards, cell migration was investigated as previously described.

Additionally, Western blot analysis was performed. After stimulation with morphine, cells were rinsed twice with ice-cold PBS, scraped and lysed with radioimmunoprecipitation assay buffer containing 150 mM NaCl, 50 mM Tris, 1% Triton X-100, 0.5% sodium deoxycholate,and 0.1% SDS, supplemented with protease inhibitors (2 g/ml aprotinin, 10 g/ml leupeptin, 1 g/ml pepstatin A, 1 mM phenylmethylsulfonylfluoride, 5 mM EDTA, 1 mM sodium orthovanadate, 10 mM sodium fluoride). Lysates were centrifuged for 30 min. Total protein concentrations were determined with the Pierce® BCA Protein Assay Kit (Thermo scientific, Rockford, USA). Samples containing 50 µg protein was boiled in SDS sample buffer (100 mM Tris/HCl (pH 6.8), 4% SDS, 0.2% bromophenol blue, 20% glycerol, 200 mM dithiothreitol) for 5 minutes and separated by 10% SDS-PAGE. Subsequently gels were semi-dry blotted on polyvinylidene difluoride membranes. After blocking with 5% non-fat dry milk, membranes were probed for the MAPK (Mitogen-activated protein kinase) expression using phospho-p44/42 MAPK (Erk 1/2), p44/42 MAPK (Erk 1/2), phospho-p38 MAPK, p38 MAPK, phospho-JNK 1/2 or JNK 1/2 (Jun NH2-Terminal Kinase), respectively (Cell Signaling Technology, Danvers, MA, USA), at a concentration of 1∶1.000 overnight at 4°C. Afterwards, the membranes were incubated with horseradish peroxidase-conjugated secondary antibody (1 ∶ 1.000) for 1 hour at room temperature and the blots were developed by chemiluminescence with 20X LumiGLO® and 20X Peroxide (CellSignaling Technology, Danvers, MA, USA). Bands were visualized with ChemiDoc™ XRS+ (Bio-RAD, USA), quantitative measurements were recorded using Image Lab (Beta 2) (Bio-RAD, USA). The expression of the phosphorylated form of each MAPK member was normalized against the expression level of its total amount. ß-Actin served as loading control.

### Statistics

All values are expressed as mean ± SEM obtained from three to five independent experiments. For the statistical analysis the unpaired t-test was performed. Differences are considered to be significant at *p≤0.05, **p≤0.01 and ***p≤0.001.

## Results

### Expression of the Opioid Receptors MOR, DOR and KOR in Oral Epithelial Cells

**Figure 1:**
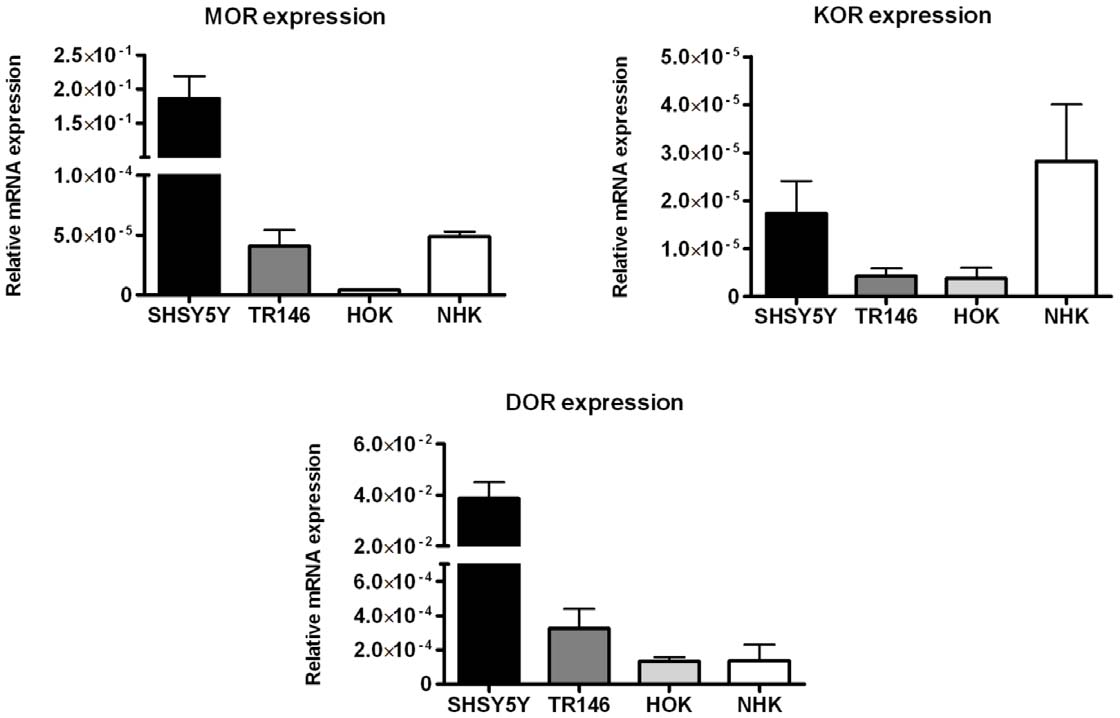
Relative mRNA expression of opioid receptors of MOR, DOR, KOR in SHSY5Y, TR146, HOK and NHK. Expression levels of KOR, MOR and DOR mRNA were normalized to the housekeeping gene YWHAZ, n = 5.

**Figure 2:**
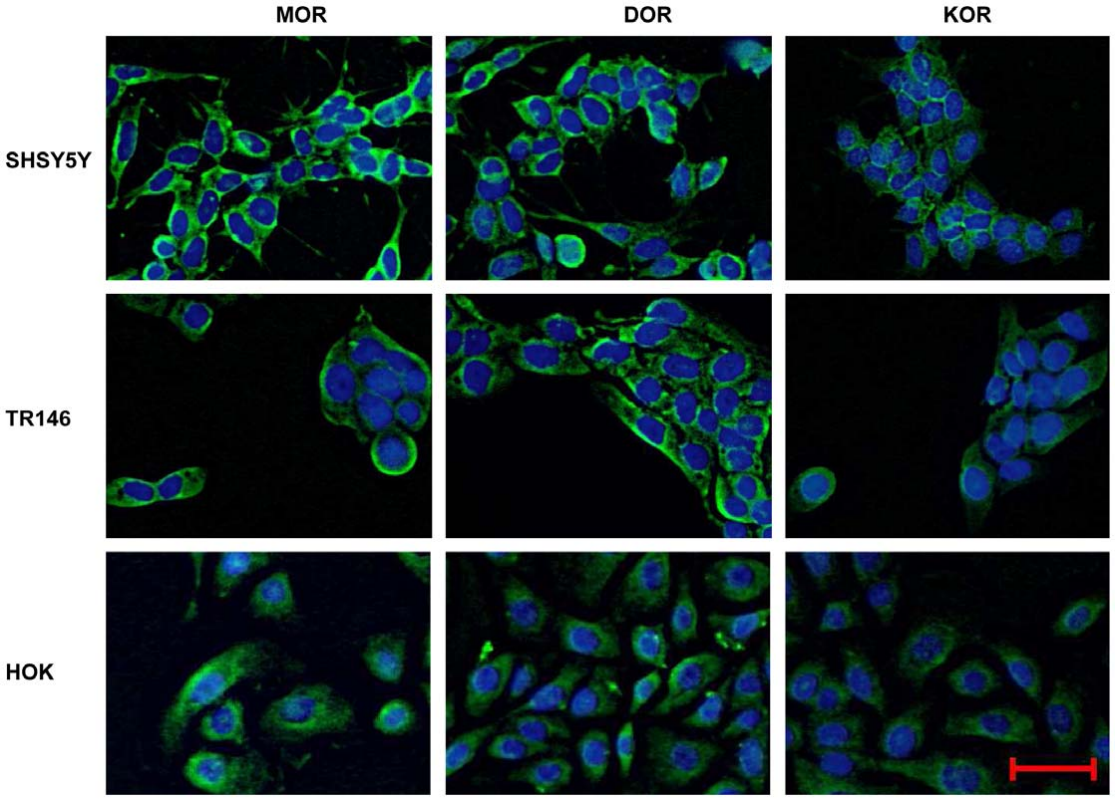
Immunostaining of MOR, DOR and KOR in SHSY5Y, TR146 and HOK. **Scale bar: 50 µm, magnification 200×.** Nuclei were stained with DAPI.

To clarify whether OR (Opioid Receptors) are present in the oral epithelium and to compare their expression to the central nervous system and to normal human keratinocytes, we determined the mRNA expression of the three OR types (MOR), (KOR) and (DOR) in the oral epithelial cell line TR146 and human oral keratinocytes (HOK). SHSY5Y and NHK served as control. In TR146 and HOK, all three opioid receptors are expressed on mRNA (Fig. 1) and protein level (Fig. 2), respectively. As expected, mRNA expression is significantly lower compared to SHSY5Y (Fig. 1). We found higher expression of DOR compared to MOR in TR146 and HOK. Surprisingly, only traces of MOR were detected in HOK (Fig. 1). The PCR products were checked by gel electrophoresis, bands were detected at 150 bp (MOR), 155 bp (DOR) and 64 bp (KOR) (data not shown).

Based on these results we decided to continue the majority of experiments with TR146 due to much easier handling and faster cell proliferation compared to HOK. Nevertheless, all experiments were repeated with HOK to ensure the transferability and consistency of the results.

### Wound Healing Assay

**Figure 3:**
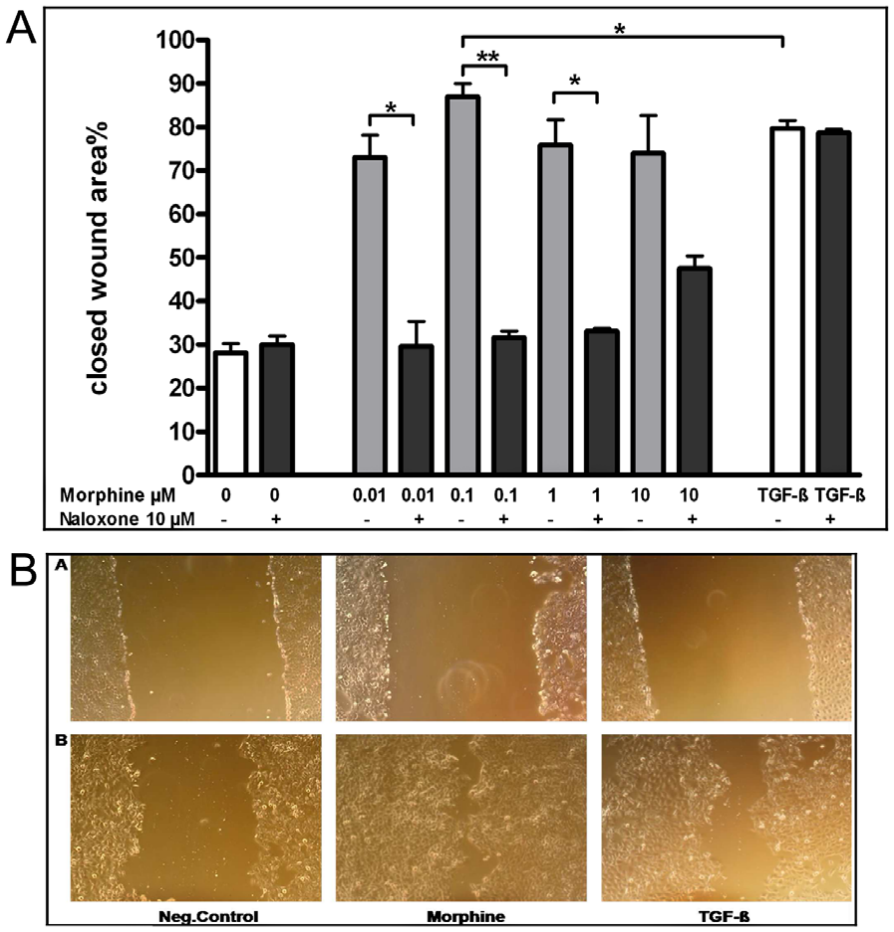
A Effect of morphine on closing an artificial ‘wound’ in TR146 cells. **Confluent cell cultures were stimulated with 0.01, 0.1, 1, and 10 µM morphine and incubated for 14 h (gray columns).** Morphine accelerated the closure of the scratch significantly compared to untreated cells (0) and TGF-ß. Pre-incubation with naloxone (10 µM) for 1 h resulted in the significant reduction of the closed wound area % (black column). *p≤0.05, **p≤0.01. **B** Representative pictures of the Scratch Assay.TR146 were stimulated with morphine (100 nM) or TGF-ß (1 ng/ml) for 14 h at 37°C. A) depicts the scratch right after wounding, B) after 14 h of incubation, n = 6.

Next we investigated the effects of morphine on the cell migration of oral epithelial cells and its ability to accelerate the closure of a ‘wound’ that has been created by scratching through a cell monolayer (scratch assay). First, we determined the impact of morphine on the cell migration (Fig. 3 A, 3 B). Clearly dose-dependent effects were observed showing particular fast migration for a morphine concentration of 100 nM (86% closed wound area versus 28% of the control). Surprisingly, this effect decreased slightly when morphine concentrations were raised up to 1 µM (75% closed wound area) and 10 µM (73% closed wound area). Thus, further experiments were conducted with a morphine concentration of 100 nM. An almost complete ‘wound closure’ was observed after 14 hours. The effect was even more pronounced than with the positive control TGF-ß (80% closed wound area; Fig. 3 A).

To investigate whether this migration enhancing effect by morphine is opioid-receptor mediated, cells were pre-incubated with the non-selective opioid receptor antagonist naloxone (10 µM) for 1 h at 37°C. Subsequently, morphine was added. The closed wound area significantly decreased, respectively, whereas no significant changes were observed for TGF-ß or the negative control (Fig. 3). Consistent results were seen with the primary oral keratinocytes, too (data not shown).

**Figure 4:**
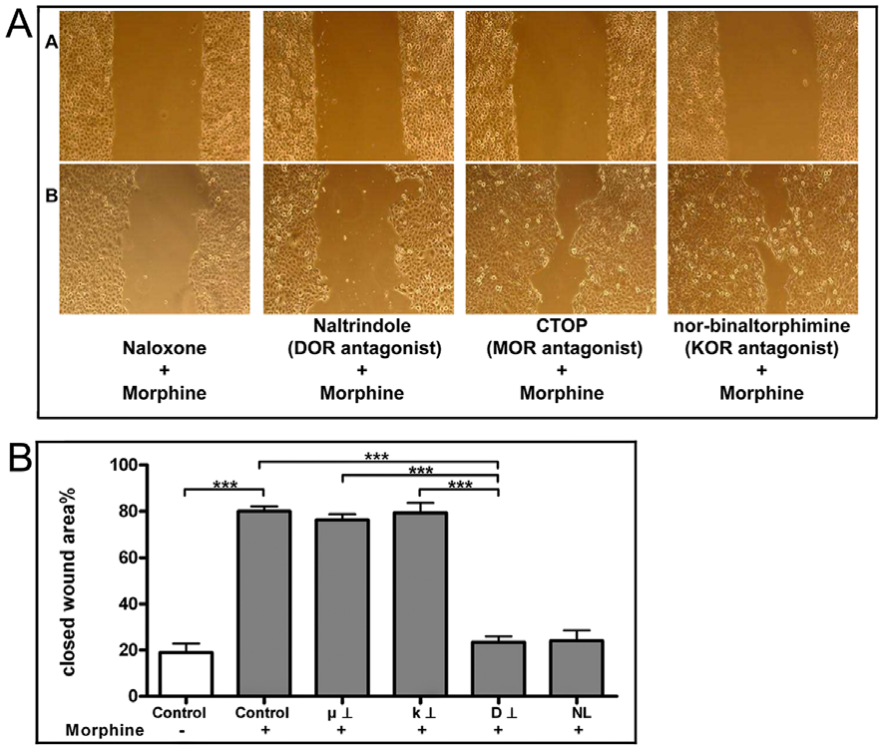
A Scratch Assay. A) depicts the scratch right after wounding, B) after 14 h of incubation. Migration enhancing effect of morphine was inhibited by naloxone and naltrindole indicating a DOR mediated effect (n = 5). **B** Effect of selective opioid receptors antagonist on wound closure ofTR146; Closed wound area % after 14 hours of treatment, pre-incubation with NL; naloxone 10 µM, μ ⊥; MOR antagonist, D ⊥; DOR antagonist, k ⊥; KOR antagonist for 1 hour prior to cell stimulation with morphine (n = 5). The OR antagonists did not show any effect on TGF-ß stimulated cell migration. ***p≤0.001.

To elucidate which receptor is responsible for the stimulation of cell migration the cells were pre-incubated with the selective DOR antagonist naltrindole (10 µM), selective MOR antagonist CTOP (10 µM) and the selective KOR antagonist nor-Binaltorphimine (10 µM). After pre-treatment following morphine application with MOR and KOR selective antagonist about 79% of the wound area was closed. In contrast, after pre-incubation with the selective DOR antagonist naltrindole only about 22% of the scratch area was closed (Fig. 4). No inhibiting effect was seen on the TGF-ß mediated cell migration (Fig. 4 B).

**Figure 5:**
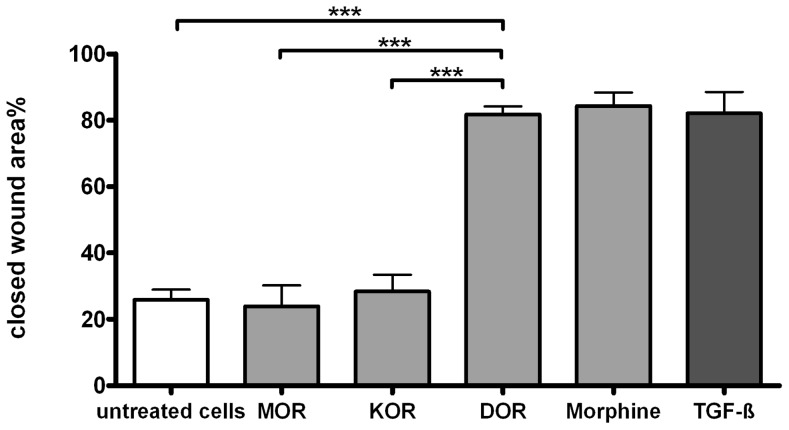
Effect of selective opioid receptor agonists on the ‘wound closure’ in TR146 cells. Closed wound area % after 14 hours of exposure with selective opioid receptor agonist for MOR (DAMGO), KOR (U-69593) and DOR (DPDPE) (100 nM) in comparison to morphine (100 nM). TGF-ß (1 ng/ml) served as a positive control, (n = 3).***p≤0.001.

The results were confirmed by using the selective opioid receptor agonists: DAMGO for MOR, DPDPE for DOR and U-69593 for KOR. The selective DOR agonist DPDPE (100 nm, 81% closed wound area) enhanced the migration to a similar degree like morphine (84%) and TGF-ß (82%). In contrast, cell stimulation with DAMGO or U-69593 did not enhance the wound closure (Fig. 5). These results were confirmed with HOK, too.

**Figure 6:**
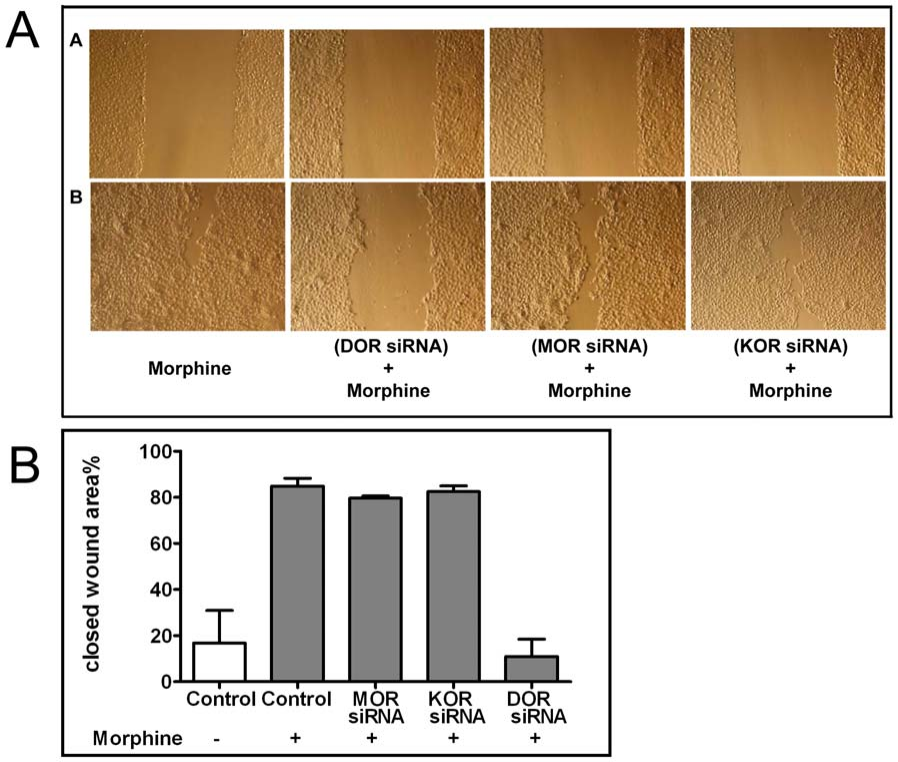
A Scratch Assay. A) depicts the scratch right after wounding, B) after 14 h of incubation. Migration enhancing effect of morphine was inhibited after knock down of DOR using siRNA. Migration was not affected after MOR and KOR knock down (n = 3). **B** Effect of siRNA knock down of opioid receptors on the ‘wound closure’; Closed wound area % after 14 hours of morphine treatment, pre-incubated with MOR siRNA, KOR siRNA and KOR siRNA (n = 3). **p≤0.01.

The key role of DOR was further substantiated by siRNA experiments. After 48 h of incubation, knock down efficiency for MOR, DOR and KOR was 74.5±6.8%, 76.3±12% and 74±7.4%, respectively. The scratch assay showed a significant reduction in the migration of DOR knock down cells (wound closure rate: 14%). In contrast, no effects were seen for MOR (wound closure rate: 80%) and KOR knock down cells (wound closure rate: 82%) (Fig. 6 A, B).

### Cell Viability and Proliferation

BrdU assay was performed in order to clarify whether the cell migration enhancing effect of morphine may be biased by a stimulation of cell proliferation. Morphine stimulated oral epithelial cells (TR146 and HOK) did not show a significant increase in cell proliferation at any concentration (data not shown). In contrast, TGF-ß exposure did result in significant increase of cell proliferation.

To ensure that morphine does not exhibit cytotoxic effects on oral epithelial cells, MTT test was performed. No cytotoxicity was found for morphine when applied in a concentration range from 1 nM up to 100 µM (data not shown).

### Determination of the Involvement of MAPK in Morphine Stimulated Cell Migration

**Figure 7:**
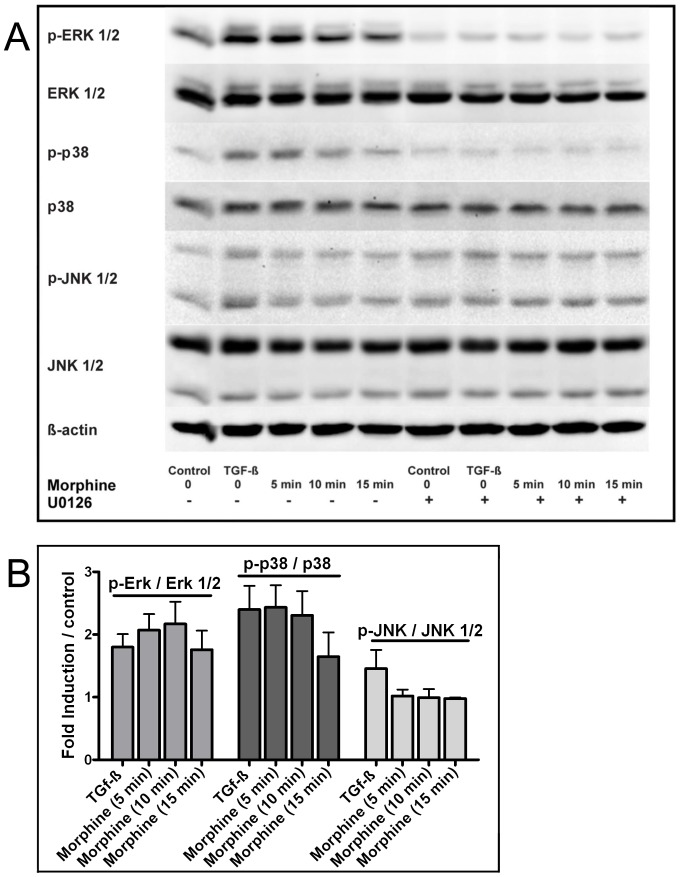
A Western blot analysis of oral epithelial cells demonstrating the effect of morphine (100 nM), TGF-ß (1 ng/ml) with and without the presence of the selective ERK 1/2 phosphorylation inhibitor U0126 (10 µM) on the phosphorylation of p38, Erk 1/2 and JNK 1/2, n = 3. **B** Relative protein expression of p-ERK 1/2, p38, and JNK 1/2 after stimulation with morphine for 5, 10, 15 min or TGF-ß for 5 min. Results are depicted as a ratio of the phosphorylated form to the total amount and normalized to the negative control, n = 3. *p≤0.05.

To elucidate the role of Erk 1/2 in oral epithelial cell migration in response to morphine stimulation, we first performed in vitro wound healing assay in the presence of the selective Erk1/2 inhibitor U0126. After 14 hours cell migration was not stimulated – the cell migration enhancing effect of morphine and TGF-ß was completely antagonized. Only 18% and 20% of the wound area was closed after treatment with morphine (100 nM) and TGF-ß (1 ng/ml) in the presences of U0126 indicating that Erk 1/2 is a crucial component of the cell migratory pathway activated by morphine and TGF-ß (data not shown). Additionally, Western blot analysis of the phosphorylated (p-) Erk 1/2, total Erk 1/2, p-p38 MAPK, p38 MAPK, p-JNK 1/2 and JNK 1/2 after stimulation with morphine and TGF-ß was performed. The results showed a time dependent increase of Erk 1/2 and p38 phosphorylation in response to morphine, whereas this effect was blocked when the cells where pre-incubated with U0126 (Fig. 7 A). The phosphorylated form of JNK1/2 did not show a significant increase with morphine but a pronounced effect with TGF-ß. Quantitative analysis showed a two fold increase of both p-Erk and p-p38 after 10 minutes stimulation with morphine (Fig. 7 B).

## Discussion

Peripheral antinociceptive effects of opioids have been first described about thirty years ago [27]. In the 1990s, evidence emerged that such effects are mediated by opioid receptors localized on peripheral sensory neurons and tissues [10]. Later on opioid receptors have also been found on immune cells such as lymphocytes, granulocytes etc. and on dermal structures such as keratinocytes and fibroblasts (for review see: [9]). The expression of opioid receptors on dermal structures is important for the cross-talk between nerves and skin in terms of analgesia, cell differentiation and migration. Furthermore, various studies describe the stimulating effects of endogenous and exogenous opioid receptor agonists on keratinocyte migration, and thus, on the formation of granulation tissue and reepithelialization of wounds [19], [20], [28]. In our study we investigated whether opioid receptors are also present on oral epithelial cells and how opioid receptor agonists influence cell behavior in terms of cell migration. These information might open up a new therapeutic rational for the treatment of oral ulcers occurring for example during oral mucositis. For this purpose we used the oral epithelial cell line TR146 which despite being isolated from a squamous cell carcinoma of the buccal mucosa expresses all natural major markers of the epithelial basal membrane and of epithelial differentiation [29]. Additionally, TR146 is also used for the construction of *in vitro* oral epithelial models [30]. To ensure the transferability of results and data, we also repeated the experiments using primary human oral keratinocytes. No differences between the cell line and the primary cells were observed.

All major opioid receptors mu-, delta-, and kappa are expressed in TR146 and primary HOK (Fig. 1 A, 1 B, 2). As expected, expression levels were lower compared to the neuroblastoma cell line SHSY5Y which served as positive control. Interestingly, we found higher mRNA levels of the DOR in the oral epithelial cells in comparison to the NHK [7], [8], [28], [31], [32], and the neuroblastoma cells which expresses MOR and DOR abundantly but KOR in traces only [33], [34]. Pain reduction in patients suffering from oral mucositis when using a morphine mouthwash [14] already indicated the involvement of peripheral opioid receptors.

Based on those results we investigated the effect of morphine on cell migration of oral epithelial cells. We opted for the use of morphine as model opioid receptor agonist as it was previously shown, that the cell migration enhancing effect of opioids is a group effect [20]. Here, we were able to demonstrate a stimulating effect of morphine on the cell migration of oral epithelial cells (Fig. 3 A, 3 B) which is a prerequisite for reepithelialization and wound closure *in vivo*. Morphine facilitated cell migration was dose dependent. Interestingly, in comparison to NHK complete ‘wound closure’ was already observed after 14 h incubation with morphine. With NHK the same effect was measurable not until 48 hours of opioid stimulation [20]. However, further increase in morphine concentration (up to 10 µM) seemed to have a reverse effect on the oral epithelial cell migration, though cell migration was still enhanced compared to the untreated control. Comparable results were described by Ohshima, et al., which observed similar effects using the epidermal growth factor EGF [35]. Other studies have demonstrated inhibition of angiogenesis by high doses of morphine and β-endorphin in a CAM assay [36] The reasons for this are ambiguous, effects of morphine on cell proliferation or cytotoxicity were excluded. Possibly it is due to nonspecific activity of excessive morphine dosages [37]. However, further studies on this are necessary.

The stimulating effect of morphine on the cell migration vanished when the oral epithelial cells were pre-incubated with the non-selective opioid receptor antagonist naloxone clearly indicating an opioid receptor mediated effect. This has also been described for other cells especially dermal cells, too [18], [20]. To clarify which opioid receptor particularly is responsible for the stimulation of cell migration we incubated the oral epithelial cells with selective opioid receptor antagonists and agonists and we knocked down the expression of MOR, KOR and DOR using RNA interference prior to the morphine application. We clearly identified DOR as the responsible receptor. Our findings are well in accordance with other studies which demonstrated delayed wound healing and hypertrophic epidermis in DOR knockout mice [38]. Thus, the key role of DOR in the wound healing process is emphasized and it can be concluded that DOR is an important player in cell differentiation and migration during wound healing.

Obviously, morphine and TGF-ß stimulate the migration of epithelial cell via similar pathways. In general, TGF-ß initiates signaling by assembling receptor complexes that activate Smad transcription factors leading to the regulation of a wide array of cellular processes such as cell growth and migration [39], [40]. TGF-ß also activates non-Smad pathways including Erk 1/2 MAPK [41], [42], JNK and p38 MAPK [43], [44]. Our results are well in accordance with these findings showing that TGF-ß increases the phosphorylation of Erk 1/2, p38 and JNK 1/2 (Fig. 7 A). For morphine, we found a time dependent activation of Erk 1/2 and p38 MAPK, but not for JNK. Erk 1/2 is crucial for the regulation of cell migration and proliferation [45], [46], [47]. p38 MAPK is important for the migration of human keratinocytes [48]. Furthermore, our studies indicate a positive cross-talk between p38 and Erk MAPK in oral epithelial as the phosphorylation of p38 was blocked after the inhibition of Erk 1/2 phosphorylation using the selective antagonist U0126 (Fig. 7 A). This has been described previously [49], [50]. Similar to oral epithelial cells, concurrent activation of Erk and p38 occurs in melanoma and the positive crosstalk between the two MAPK members stimulates cell migration and proliferation [51]. Although more studies are required for a full understanding of the exact signaling mechanisms underlying morphine stimulated cell migration, this study definitely shows the involvement of the MAPK members Erk 1/2 and p38.

In conclusion, our findings might open up a new therapeutic rational for the treatment of patients with chemo-/radiotherapy-induced oral mucositis. The basis for this is the presence of ORs on the oral epithelium. A local application of opioids can allow for efficient pain reduction, facilitated wound healing and wound closure due to the stimulation of cell migration. Definitely, further studies are needed, especially in more complex *in vitro* and *in vivo* systems. Nevertheless, morphine could be an effective and safe therapeutic option for oral wounds.

## References

[1] Bellm LA, Epstein JB, Rose-Ped A, Martin P, Fuchs HJ (2000) Patient reports of complications of bone marrow transplantation. Support Care Cancer 8: 33–39. 10650895 10.1007/s005209900095

[2] Silvermann S Jr (2007) Diagnosis and management of oral mucositis. J Support Oncol 5: 13–21. 17366929

[3] Sonis ST (2007) Pathobiology of oral mucositis: novel insights and opportunities. J Support Oncol 5: 3–11. 18046993

[4] Stone R, Fliedner MC, Smiet AC (2005) Management of oral mucositis in patients with cancer. Eur J Oncol Nurs 9 Suppl 1: S24–32. 16202654 10.1016/j.ejon.2005.08.004

[5] Stein C (2003) Opioid receptors on peripheral sensory neurons. Adv Exp Med Biol 521: 69–76. 12617565

[6] Stein C, Schafer M, Machelska H (2003) Attacking pain at its source: new perspectives on opioids. Nat Med 9: 1003–1008. 12894165 10.1038/nm908

[7] Bigliardi PL, Bigliardi-Qi M, Buechner S, Rufli T (1998) Expression of mu-opiate receptor in human epidermis and keratinocytes. J Invest Dermatol 111: 297–301. 9699733 10.1046/j.1523-1747.1998.00259.x

[8] Bigliardi-Qi M, Sumanovski LT, Buchner S, Rufli T, Bigliardi PL (2004) Mu-opiate receptor and Beta-endorphin expression in nerve endings and keratinocytes in human skin. Dermatology 209: 183–189. 15459530 10.1159/000079887

[9] Rachinger-Adam B, Conzen P, Azad SC (2011) Pharmacology of peripheral opioid receptors. Curr Opin Anaesthesiol 24: 408–413. 21659869 10.1097/ACO.0b013e32834873e5

[10] Stein C (1993) Peripheral mechanisms of opioid analgesia. Anesth Analg 76: 182–191. 8380316 10.1213/00000539-199301000-00031

[11] Schäfer M, Imai Y, Uhl GR, Stein C (1995) Inflammation enhances peripheral mu-opioid receptor-mediated analgesia, but not mu-opioid receptor transcription in dorsal root ganglia. Eur J Pharmacol 279: 165–169. 7556397 10.1016/0014-2999(95)00150-j

[12] Stein C (1995) The control of pain in peripheral tissue by opioids. N Engl J Med 332: 1685–1690. 7760870 10.1056/NEJM199506223322506

[13] Stein C, Hassan AH, Przewlocki R, Gramsch C, Peter K, et al (1990) Opioids from immunocytes interact with receptors on sensory nerves to inhibit nociception in inflammation. Proc Natl Acad Sci U S A 87: 5935–5939. 1974052 10.1073/pnas.87.15.5935 PMC54444

[14] Cerchietti LC, Navigante AH, Bonomi MR, Zaderajko MA, Menendez PR, et al (2002) Effect of topical morphine for mucositis-associated pain following concomitant chemoradiotherapy for head and neck carcinoma. Cancer 95: 2230–2236. 12412178 10.1002/cncr.10938

[15] Flock P (2003) Pilot study to determine the effectiveness of diamorphine gel to control pressure ulcer pain. J Pain Symptom Manage 25: 547–554. 12782435 10.1016/s0885-3924(03)00140-4

[16] LeBon B, Zeppetella G, Higginson IJ (2009) Effectiveness of topical administration of opioids in palliative care: a systematic review. J Pain Symptom Manage 37: 913–917. 19321297 10.1016/j.jpainsymman.2008.06.007

[17] Platzer M, Likar R, Stein C, Beubler E, Sittl R (2005) [Topical application of morphine gel in inflammatory mucosal and cutaneous lesions]. Schmerz 19: 296–301. 15568159 10.1007/s00482-004-0372-9

[18] Poonawala T, Levay-Young BK, Hebbel RP, Gupta K (2005) Opioids heal ischemic wounds in the rat. Wound Repair Regen 13: 165–174. 15828941 10.1111/j.1067-1927.2005.130207.x

[19] Küchler S, Wolf NB, Heilmann S, Weindl G, Helfmann J, et al (2010) 3D-wound healing model: influence of morphine and solid lipid nanoparticles. J Biotechnol 148: 24–30. 20138929 10.1016/j.jbiotec.2010.01.001

[20] Wolf NB, Küchler S, Radowski MR, Blaschke T, Kramer KD, et al (2009) Influences of opioids and nanoparticles on in vitro wound healing models. Eur J Pharm Biopharm 73: 34–42. 19344759 10.1016/j.ejpb.2009.03.009

[21] Cerchietti LC, Navigante AH, Korte MW, Cohen AM, Quiroga PN, et al (2003) Potential utility of the peripheral analgesic properties of morphine in stomatitis-related pain: a pilot study. Pain 105: 265–273. 14499444 10.1016/s0304-3959(03)00227-6

[22] Gysler A, Lange K, Korting HC, Schäfer-Korting M (1997) Prednicarbate biotransformation in human foreskin keratinocytes and fibroblasts. Pharm Res 14: 793–797. 9210199 10.1023/a:1012162708675

[23] O'Kane S, Ferguson MW (1997) Transforming growth factor beta s and wound healing. Int J Biochem Cell Biol 29: 63–78. 9076942 10.1016/s1357-2725(96)00120-3

[24] Geback T, Schulz MM, Koumoutsakos P, Detmar M (2009) TScratch: a novel and simple software tool for automated analysis of monolayer wound healing assays. Biotechniques 46: 265–274. 19450233 10.2144/000113083

[25] Mosmann T (1983) Rapid colorimetric assay for cellular growth and survival: application to proliferation and cytotoxicity assays. J Immunol Methods 65: 55–63. 6606682 10.1016/0022-1759(83)90303-4

[26] Favata MF, Horiuchi KY, Manos EJ, Daulerio AJ, Stradley DA, et al (1998) Identification of a novel inhibitor of mitogen-activated protein kinase kinase. J Biol Chem 273: 18623–18632. 9660836 10.1074/jbc.273.29.18623

[27] Ferreira SH, Nakamura M (1979) II - Prostaglandin hyperalgesia: the peripheral analgesic activity of morphine, enkephalins and opioid antagonists. Prostaglandins 18: 191–200. 230543 10.1016/0090-6980(79)90104-7

[28] Bigliardi PL, Tobin DJ, Gaveriaux-Ruff C, Bigliardi-Qi M (2009) Opioids and the skin–where do we stand? Exp Dermatol 18: 424–430. 19382313 10.1111/j.1600-0625.2009.00844.x

[29] Rupniak HT, Rowlatt C, Lane EB, Steele JG, Trejdosiewicz LK, et al (1985) Characteristics of four new human cell lines derived from squamous cell carcinomas of the head and neck. J Natl Cancer Inst 75: 621–635. 2413234

[30] Moharamzadeh K, Brook IM, Van Noort R, Scutt AM, Thornhill MH (2007) Tissue-engineered oral mucosa: a review of the scientific literature. J Dent Res 86: 115–124. 17251509 10.1177/154405910708600203

[31] Cheng B, Liu HW, Fu XB, Sheng ZY, Li JF (2008) Coexistence and upregulation of three types of opioid receptors, mu, delta and kappa, in human hypertrophic scars. Br J Dermatol 158: 713–720. 18284397 10.1111/j.1365-2133.2008.08449.x

[32] Salemi S, Aeschlimann A, Reisch N, Jungel A, Gay RE, et al (2005) Detection of kappa and delta opioid receptors in skin–outside the nervous system. Biochem Biophys Res Commun 338: 1012–1017. 16263089 10.1016/j.bbrc.2005.10.072

[33] Prather PL, Tsai AW, Law PY (1994) Mu and delta opioid receptor desensitization in undifferentiated human neuroblastoma SHSY5Y cells. J Pharmacol Exp Ther 270: 177–184. 8035314

[34] Zadina JE, Harrison LM, Ge LJ, Kastin AJ, Chang SL (1994) Differential regulation of mu and delta opiate receptors by morphine, selective agonists and antagonists and differentiating agents in SH-SY5Y human neuroblastoma cells. J Pharmacol Exp Ther 270: 1086–1096. 7932156

[35] Ohshima M, Sato M, Ishikawa M, Maeno M, Otsuka K (2002) Physiologic levels of epidermal growth factor in saliva stimulate cell migration of an oral epithelial cell line, HO-1-N-1. Eur J Oral Sci 110: 130–136. 12013556 10.1034/j.1600-0722.2002.11179.x

[36] Pasi A, Qu BX, Steiner R, Senn HJ, Bar W, et al (1991) Angiogenesis: modulation with opioids. Gen Pharmacol 22: 1077–1079. 1725773 10.1016/0306-3623(91)90580-y

[37] Farooqui M, Li Y, Rogers T, Poonawala T, Griffin RJ, et al (2007) COX-2 inhibitor celecoxib prevents chronic morphine-induced promotion of angiogenesis, tumour growth, metastasis and mortality, without compromising analgesia. Br J Cancer 97: 1523–1531. 17971769 10.1038/sj.bjc.6604057 PMC2360252

[38] Bigliardi-Qi M, Gaveriaux-Ruff C, Zhou H, Hell C, Bady P, et al (2006) Deletion of delta-opioid receptor in mice alters skin differentiation and delays wound healing. Differentiation 74: 174–185. 16683988 10.1111/j.1432-0436.2006.00065.x

[39] Massague J (1998) TGF-beta signal transduction. Annu Rev Biochem 67: 753–791. 9759503 10.1146/annurev.biochem.67.1.753

[40] Massague J, Chen YG (2000) Controlling TGF-beta signaling. Genes Dev 14: 627–644. 10733523

[41] Yue J, Frey RS, Mulder KM (1999) Cross-talk between the Smad1 and Ras/MEK signaling pathways for TGFbeta. Oncogene 18: 2033–2037. 10208426 10.1038/sj.onc.1202521

[42] Hartsough MT, Mulder KM (1995) Transforming growth factor beta activation of p44mapk in proliferating cultures of epithelial cells. J Biol Chem 270: 7117–7124. 7706248 10.1074/jbc.270.13.7117

[43] Weston CR, Davis RJ (2007) The JNK signal transduction pathway. Curr Opin Cell Biol 19: 142–149. 17303404 10.1016/j.ceb.2007.02.001

[44] Zhang YE (2009) Non-Smad pathways in TGF-beta signaling. Cell Res 19: 128–139. 19114990 10.1038/cr.2008.328 PMC2635127

[45] Chen Z, Gibson TB, Robinson F, Silvestro L, Pearson G, et al (2001) MAP kinases. Chem Rev 101: 2449–2476. 11749383 10.1021/cr000241p

[46] Huang C, Jacobson K, Schaller MD (2004) MAP kinases and cell migration. J Cell Sci 117: 4619–4628. 15371522 10.1242/jcs.01481

[47] Pearson G, Robinson F, Beers Gibson T, Xu BE, Karandikar M, et al (2001) Mitogen-activated protein (MAP) kinase pathways: regulation and physiological functions. Endocr Rev 22: 153–183. 11294822 10.1210/edrv.22.2.0428

[48] Li W, Nadelman C, Henry G, Fan J, Muellenhoff M, et al (2001) The p38-MAPK/SAPK pathway is required for human keratinocyte migration on dermal collagen. J Invest Dermatol 117: 1601–1611. 11886529 10.1046/j.0022-202x.2001.01608.x

[49] Moon SE, Bhagavathula N, Varani J (2002) Keratinocyte stimulation of matrix metalloproteinase-1 production and proliferation in fibroblasts: regulation through mitogen-activated protein kinase signalling events. Br J Cancer 87: 457–464. 12177784 10.1038/sj.bjc.6600478 PMC2376127

[50] Ibrahim AS, El-Remessy AB, Matragoon S, Zhang W, Patel Y, et al Retinal microglial activation and inflammation induced by amadori-glycated albumin in a rat model of diabetes. Diabetes 60: 1122–1133. 10.2337/db10-1160 PMC3064086 21317295

[51] Estrada Y, Dong J, Ossowski L (2009) Positive crosstalk between ERK and p38 in melanoma stimulates migration and in vivo proliferation. Pigment Cell Melanoma Res 22: 66–76. 18983537 10.1111/j.1755-148X.2008.00520.x

